# Pregnancy Outcome and Postnatal Chromosome Analysis of the Cord Blood and Chorionic Villi in Two Cases after Intrauterine Transfer of Mosaic Aneuploid Blastocysts

**DOI:** 10.1155/2022/1763948

**Published:** 2022-07-19

**Authors:** Yuki Ito, Taizan Kamide, Kosuke Taniguchi, Taisuke Sato, Michihiro Yamamura, Akiko Konishi, Ken Takahashi, Hiroshi Kishi, Kenichiro Hata, Osamu Samura, Aikou Okamoto

**Affiliations:** ^1^Department of Obstetrics and Gynaecology, The Jikei University School of Medicine, 3-25-8, Nishi-Shinbashi, Minato-ku, Tokyo, Japan 105-8461; ^2^Department of Maternal-Fetal Biology, National Research Institute for Child Health and Development, 2-10-1, Okura, Setagaya-ku, Tokyo 157-8535, Japan

## Abstract

The long-term prognosis and genetic mechanism of pregnancy after intrauterine mosaic aneuploid blastocyst transfer remain unknown. We report the case of two babies after the aforementioned procedure and chromosomal analysis of their cord blood and chorionic villi. *Case Report 1*. A 41-year-old primipara, with two previous spontaneous abortions, was pregnant after intrauterine transfer of a blastocyst carrying 40% mosaicism of long-arm monosomy of chromosome 5. The amniocentesis results were 46,XX. A cesarean section was performed at 39 weeks. The female infant was 3,315 g at birth. *Case Report 2*. A 44-year-old primipara, with two spontaneous abortions, was pregnant after intrauterine transfer of a blastocyst carrying 40% mosaicism of long-arm monosomy of chromosome 9 and monosomy of chromosome 14. After genetic counselling, she decided not to undergo amniocentesis. No abnormalities were found by ultrasound. A cesarean section was performed at 38 weeks. The male infant was 3,340 g at birth. Chromosome analyses of postnatal cord blood and chorionic villi were performed using SNP arrays. The cord blood and chorionic villi showed no chromosomal structural abnormalities or mosaicism. For both, no disorders were observed at 10 months of age. We experienced the birth of babies after intrauterine transfer of mosaic aneuploid blastocysts.

## 1. Introduction

In preimplantation genetic testing for aneuploidy (PGT-A), approximately five cells are taken from the trophectoderm (TE) of the blastocyst. After whole genome amplification, next-generation sequencing (NGS) or array comparative genomic hybridization (aCGH) can be used to diagnose aneuploidy. Frequently, mosaic embryos are detected by PGT-A test results [1]. The transfer of euploid embryos is the first priority in preimplantation diagnosis, but the transfer of mosaic embryos may be considered if euploid embryos cannot be obtained [2].

The clinical results after the transfer of mosaic embryos are less favourable than those after the transfer of euploid embryos, with an implantation rate of approximately 40%–50% and an ongoing pregnancy rate of approximately 30%–40% [3]. In addition, the higher the mosaic ratio, the lower the pregnancy rate, the higher the miscarriage rate, and the lower the ongoing pregnancy rate.

There are reports of live births following mosaic embryo transfer. Victor et al. reported 21 live births after mosaic embryo transfer, 14 of which underwent amniocentesis: 11 with a normal karyotype, 2 with microdeletions, and 1 with a balanced translocation [4]. When mosaic embryos are transferred, there is a possibility of confined placental mosaicism, true foetal mosaicism with mosaicism in the foetus, or uniparental disomy (UPD)/loss of heterozygosity (LOH). Therefore, chromosome analysis of both the foetus and placenta in cases of pregnancies after mosaic embryo transfer is important.

We report the postnatal chromosome analysis of the cord blood and chorionic villi of two babies following mosaic embryo transfer.

## 2. Case Presentation of Cases

### 2.1. Case 1

**Figure 1:**
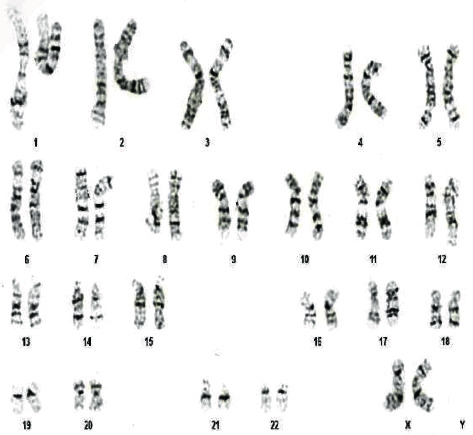
Normal female karyotype obtained after amniocentesis at 17 weeks and 1 d of pregnancy in Case 1.

A primipara had two previous spontaneous abortions. At the age of 40 years and 3 months, she had her eggs retrieved. TE biopsies were carried out on 3 blastocysts for PGT-A. aCGH revealed one mosaic embryo and no euploid embryo. Although she continued egg retrievals, she could not obtain a euploid embryo. At the age of 41 years and 5 months, she became pregnant after intrauterine blastocyst transfer with 40% mosaicism of the entire long-arm monosomy of chromosome 5 at an in vitro fertilization (IVF) clinic. She had no previous medical history. There was no family history of congenital disorder or genetic disorder. The couple were nonconsanguineous. The patient was referred to our hospital at 11 weeks of pregnancy. At 15 weeks of pregnancy, genetic counselling was provided to the couple. After counselling, she requested amniocentesis, which was performed at 17 weeks and 1 day of pregnancy. The amniocentesis (G-banding) results were 46,XX (15 cells) (Figure 1) (Table 1). Foetal screening ultrasonography at 20 and 30 weeks' gestation did not reveal any abnormalities. She underwent a cesarean section due to labour arrest at 39 weeks and 3 d of gestation. The female infant had a weight of 3,315 g, an Apgar score of 8/9 (1 min/5 min), and no evident abnormalities at birth. The size of the main placenta (placenta 1) was 18 × 17 × 1.5 cm^3^ with a 7 × 5 × 1.5 cm^3^ succenturiate placenta (placenta 2). No motor and intellectual disorders were observed at 10 months of age. Height and weight were appropriate for age.

### 2.2. Case 2

A primipara had two previous spontaneous abortions. At the age of 43 years and 8 months, she had her eggs retrieved at an IVF clinic. TE biopsies were carried out on one blastocyst for PGT-A. NGS revealed one mosaic embryo. At the age of 44 years and 1 month, she became pregnant after intrauterine blastocyst transfer with 40% mosaicism of long-arm monosomy of chromosome 9 and monosomy of chromosome 14. She had a history of myomectomy at the age of 39 years. There was no family history of congenital disorder or genetic disorder. The couple were nonconsanguineous. The patient was referred to our hospital at 10 weeks of pregnancy. At 12 weeks of pregnancy, genetic counselling was provided to the couple. She strongly desired to continue the pregnancy and hoped that amniocentesis would not be performed if ultrasonography did not show an abnormal foetal morphology (Table 1). Foetal screening ultrasonography at 12, 20, and 30 weeks' gestation did not reveal any abnormalities. A cesarean section was performed at 38 weeks and 3 d. The female infant had a weight of 3,340 g, an Apgar score of 8/9 (1 min/5 min), and no evident abnormalities at birth. The placenta showed no gross abnormalities. No motor and intellectual disorders were observed 1 year after birth.

### 2.3. Genome-Wide Single-Nucleotide Polymorphism Array

**Figure 2:**
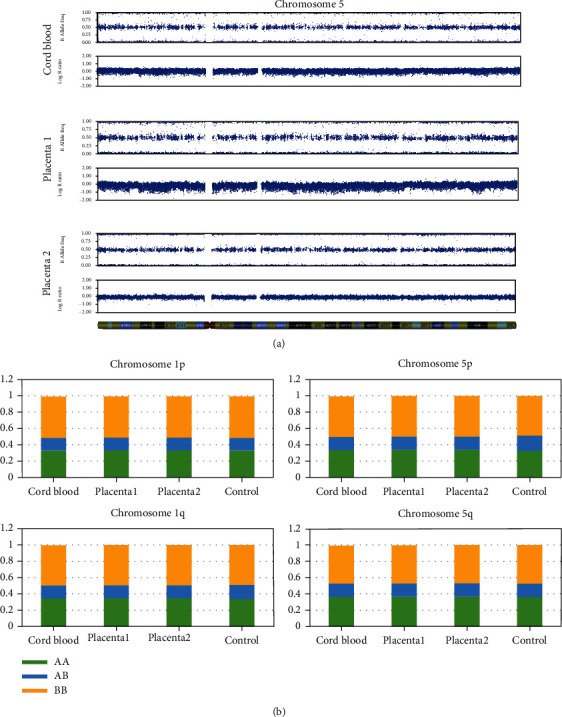
(a) Results of single-nucleotide polymorphism array analysis of the umbilical cord blood, main placenta (placenta 1), and succenturiate placenta (placenta 2) in Case 1: the B allele frequency indicates the frequency of B allele. The logR ratio indicates the copy number. No monosomy mosaicism or loss of heterozygosity was observed on the long arm of chromosome 5. (b) Proportion of single-nucleotide polymorphisms (AA, AB, and BB). There was no increase in the percentage of AA and BB compared to the control sample (peripheral blood of an adult woman with no previous medical history) analysed in the same panel. Chromosome 5 had similar polymorphism ratios compared to chromosome 1.

**Figure 3:**
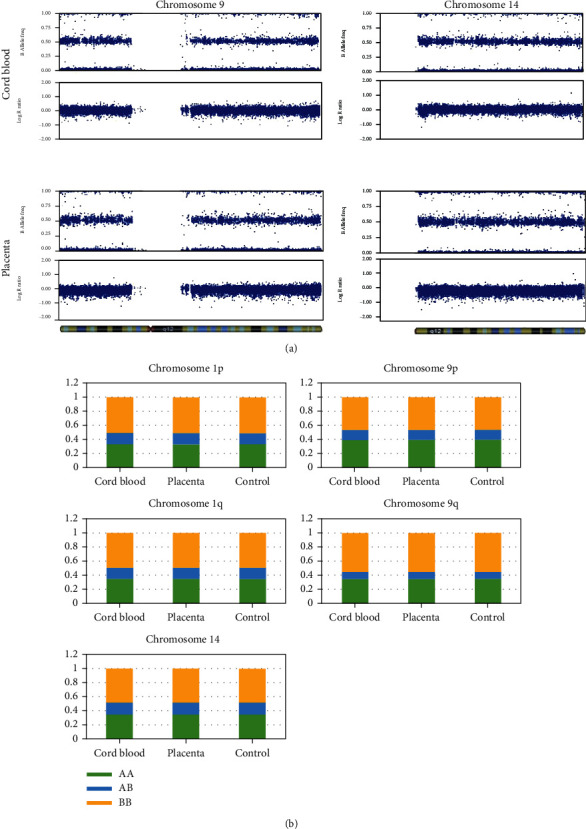
(a) Results of single-nucleotide polymorphism array analysis of the umbilical cord blood and placenta in Case 2: no monosomy mosaicism or loss of heterozygosity was observed on the long arm of chromosome 9 and chromosome 14. (b) Proportion of single-nucleotide polymorphisms (AA, AB, and BB) in Case 2. There was no increase in the percentage of AA and BB compared to that in the control sample (peripheral blood of an adult woman with no previous medical history) analysed in the same panel. Chromosomes 9 and 14 had similar polymorphism ratios compared to chromosome 1.

DNA was extracted from the umbilical cord blood sample and from the chorionic villi sample (Case 1: placentas 1 and 2, Case 2: placenta) as previously described [5]. Genome-wide single-nucleotide polymorphism (SNP) array analysis of the DNA samples was performed using the Infinium Asian Screening Array-24 v1.0 BeadChip (Illumina, San Diego, CA, USA) according to the manufacturer's protocols. The SNP arrays were scanned using the Illumina iScan system, and the data were analysed with KaryoStudio (version 1.4, Illumina, San Diego, CA, USA) using the default cnvPartition algorithm settings (version 3.0.7). Each SNP is sorted into A allele and B allele according to Illumina's protocol (Illumina, San Diego, CA, USA). The B allele frequency indicates the ratio of B allele. The logR ratio indicates the copy number. No decrease in the logR ratio was observed in the long arm of chromosome 5 of Case 1 and the long arm of chromosome 9 or chromosome 14 of Case 2. There was no mosaicism or LOH in B allele frequency (Figures 2 and 3). The proportion of SNP (AA, AB, and BB) was calculated, but there was no increase in the percentage of AA and BB compared to that in the control sample (peripheral blood of an adult woman with no previous medical history) analysed in the same panel. The proportion of SNP of chromosome 5 of Case 1 and chromosome 9 or chromosome 14 of Case 2 was comparable to that of chromosome 1. These chromosomes had similar polymorphism ratios compared to chromosome 1 (Figures 2 and 3). Thus, it was concluded that no mosaicism could be detected by the SNP array in both cases. There were no findings of structural abnormalities in other chromosomes.

This study was approved by the Institutional Review Boards (IRBs) of the National Center for Child Health and Development and the Jikei University School of Medicine (IRB number: 234 and IRB number: 27-060 (7945), respectively). Written informed consent was obtained from all subjects for the publication of this case report and the accompanying images.

## 3. Discussion

Chromosome analysis of cord blood and chorionic villi of two babies obtained after mosaic embryo transfer showed no abnormalities in the chromosome structure or mosaicism.

SNP arrays have the advantages of analysing a large number of cells simultaneously, cells in all cell cycles, and specimens without culturing cells [6]. UPD/LOH can also be detected in SNP arrays. There are reports showing the SNP array being more sensitive than aCGH and able to detect more than 5% of mosaicism [6, 7].

Partial deletion of the long arm of chromosome 5 has been reported to cause foetal growth restriction, duodenal obstruction, and cardiac disease [8]. Long-arm monosomy of chromosome 9 indicates foetal mortality. Partial deletion of the long arm of chromosome 9 has been reported to cause foetal hydrops, severe developmental delay, cardiac disease, and respiratory failure [9]. Partial deletion of the long arm of chromosome 14 has been associated with holoprosencephaly, microcephaly, and facial malformations [10]. There was no abnormality found in both infants.

Chromosome analysis of the entire placental tissue was difficult, which is a limitation of this study. However, as far as we could analyse, in both Cases 1 and 2, the SNP array results were normal, and there were no findings of LOH for whole chromosomes. These results can be attributed to two possibilities. First, once fertilization occurs normally, the monosomy mosaic appears during somatic cell division but decreases or disappears during development. Embryos with segmental mosaicism have the same miscarriage rate and live birth rate as the control group with a normal karyotype, because segment abnormal cells lacking centromeres cannot attach to the spindle during mitosis and do not proliferate during cell division [1]. Second is the presence of false positives during PGT-A analysis [11].

Mosaic embryos may be false positives for PGT-A analysis, or aneuploid cells may disappear during cell division. However, when mosaic embryos are transferred, there is a possibility of confined placental mosaicism, true foetal mosaicism, or UPD/LOH. Confined placental mosaicism may affect the placental function and cause intrauterine foetal growth restriction or preterm birth, even if no abnormalities are detected in the foetus [12]. In true foetal mosaicism, depending on the mosaic ratio and chromosome position, foetal morphological abnormalities and foetal growth may be detected [6]. Indeed, in one case, the same mosaicism observed on PGT-A has been reported in infants after birth [13]. Therefore, anxiety often persists during ongoing pregnancy, even when adequate genetic counselling is provided prior to mosaic embryo transfer. Currently, the number of reports on mosaic embryo transfer is increasing, but there are not enough reported cases to evaluate the effects of each specific mosaic composition and mosaicism rate on the pregnancy outcome and foetus. Therefore, it is important to continue to accumulate detailed clinical and genetic data on mosaic embryo transfers in the future.

In conclusion, we performed postnatal chromosome analysis of the cord blood and chorionic villi of two babies after mosaic embryo transfer. Our results may provide useful information for genetic counselling before embryo transfer and prenatal testing.

## Figures and Tables

**Table 1:** Summary of Cases 1 and 2.

	Age at egg retrieval (years)	Age at embryo transplantation (years)	PGT-A		Amniocentesis	Postnatal chromosomal analysis	
			Chromosomal constitution	Mosaic percentage (%)		Placenta	Cord blood
Case 1	40	41	Monosomy 5q (aCGH)	40	46,XX (G-banding)	46,XX (SNP array)	46,XX (SNP array)
Case 2	43	44	Monosomy 9q (NGS)	40	Not performed	46,XX (SNP array)	46,XX (SNP array)
			Monosomy 14 (NGS)	40			

PGT-A = preimplantation genetic testing for aneuploidy; aCGH = array comparative genomic hybridization; NGS = next-generation sequencing; SNP = single-nucleotide polymorphism.

## Data Availability

All data generated or analysed during this study are included in this article.
